# Diversity of 23S rRNA Genes within Individual Prokaryotic Genomes

**DOI:** 10.1371/journal.pone.0005437

**Published:** 2009-05-05

**Authors:** Anna Pei, Carlos W. Nossa, Pooja Chokshi, Martin J. Blaser, Liying Yang, David M. Rosmarin, Zhiheng Pei

**Affiliations:** 1 Washington University, College of Arts and Sciences, St. Louis, Missouri, United States of America; 2 Department of Medicine, New York University School of Medicine, New York, New York, United States of America; 3 Tufts University College of Arts and Sciences, Medford, Massachusetts, United States of America; 4 Department of Microbiology, New York University School of Medicine, New York, New York, United States of America; 5 Department of Veterans Affairs, New York Harbor Healthcare System, New York, New York, United States of America; 6 Department of Pathology, New York University School of Medicine, New York, New York, United States of America; University of California, Berkeley, United States of America

## Abstract

**Background:**

The concept of ribosomal constraints on rRNA genes is deduced primarily based on the comparison of consensus rRNA sequences between closely related species, but recent advances in whole-genome sequencing allow evaluation of this concept within organisms with multiple rRNA operons.

**Methodology/Principal Findings:**

Using the 23S rRNA gene as an example, we analyzed the diversity among individual rRNA genes within a genome. Of 184 prokaryotic species containing multiple 23S rRNA genes, diversity was observed in 113 (61.4%) genomes (mean 0.40%, range 0.01%–4.04%). Significant (1.17%–4.04%) intragenomic variation was found in 8 species. In 5 of the 8 species, the diversity in the primary structure had only minimal effect on the secondary structure (stem versus loop transition). In the remaining 3 species, the diversity significantly altered local secondary structure, but the alteration appears minimized through complex rearrangement. Intervening sequences (IVS), ranging between 9 and 1471 nt in size, were found in 7 species. IVS in *Deinococcus radiodurans* and *Nostoc sp.* encode transposases. *T. tengcongensis* was the only species in which intragenomic diversity >3% was observed among 4 paralogous 23S rRNA genes.

**Conclusions/Significance:**

These findings indicate tight ribosomal constraints on individual 23S rRNA genes within a genome. Although classification using primary 23S rRNA sequences could be erroneous, significant diversity among paralogous 23S rRNA genes was observed only once in the 184 species analyzed, indicating little overall impact on the mainstream of 23S rRNA gene-based prokaryotic taxonomy.

## Introduction

Ribosomes are the large, ribonucleoprotein machinery in which proteins are synthesized. The structure of ribosomes is largely conserved amongst the three kingdoms of life. In prokaryotes, the ribosome is composed of two subunits: a large 50S subunit, and a small 30S subunit. The 50S subunit contains a 23S, a 5S rRNA, and more than 30 proteins. The 30S subunit contains a 16S rRNA plus 20 proteins. Precise spatial relationships may be essential for assembly of functional ribosomes, constraining ribosomal RNA genes from drastic change [1].

Because of functional constraints on the structure of rRNA gene sequences, certain regions in rRNA genes that are in contact with other components in the ribosome are conserved, while sequences between the conserved regions mutate at faster rates. The conserved regions are useful for determining distant relationships, while the “fast” regions are useful for distinguishing closely-related organisms. Horizontal gene transfer events that could falsify the evolutionary history of an organism are unlikely to occur in the highly constrained rRNA genes[2], [3], [4]. These features make rRNA genes the most suitable molecular chronometer for both phylogenetic analysis and taxonomic classification of cellular organisms [5].

In general, 16S and 23S rRNA based phylogenetic trees are in good agreement[6], [7], while 5S rRNA is considered to not contain sufficiently long sequences for statistically significant comparisons. The detailed and comprehensive phylogenies inferred from 16S as well as 23S rRNA sequence comparisons provide three primary domains [5], [8] the Bacteria, Archaea, and Eukarya. However, 23S rRNA has lost favor to 16S rRNA in phylogenetic analysis and is rarely used in taxonomic classification because of the lack of established broad-range sequencing primers and the difficulty of sequencing larger genes with early sequencing technology.

There has been renewed interest in the use of the 23S rRNA gene, driven by the decrease in sequencing costs with next generation DNA sequencing technology (454 sequencing) and demand from the new Roadmap Initiative in the Human Microbiome Project (http://nihroadmap.nih.gov/hmp/). Compared to 16S rRNA genes, 23S rRNA genes contain more characteristic sequence stretches due to a greater length, unique insertions and/or deletions, and possibly better phylogenetic resolution because of higher sequence variation [9]. A recent study indicated that 23S rRNA genes also contain conserved regions for designing broad-range primers with a similar degree of universality to the broad-range primers for 16S rRNA genes [10].

Gene redundancy is uncommon in prokaryotic genomes, yet the rRNA genes can vary from one to as many as 15 copies in a single genome [11]. Divergent evolution between rRNA genes in the same genome may corrupt the record of evolutionary history and obscure the true identity of an organism. When substantial variation occurs, use of rRNA gene sequences may lead to the artificial classification of an organism into more than one species. In this study, we performed a systematic survey of intragenomic variation of 23S rRNA genes in genomes representing 184 prokaryotic species.

## Materials and Methods

### Annotation of 23S rRNA genes

Gene sequences were from the Complete Microbial Genomes database at the NCBI website (http://www.ncbi.nlm.nih.gov/genomes/MICROBES/Complete.html). For some species, more than one genome was available. Comparison of homologous 23S rRNA genes between these genomes did not show >3% sequence difference (data not shown). To avoid duplicating any species, only the most completely annotated genome was included for analysis. Lengths of 23S rRNA genes were identified by using experimentally defined 23S rRNA sequences from the closest relatives available and verified by 2° structure analysis based on minimizing free energy, using RNAstructure [12] and Rnaviz [13], with experimentally defined 23S rRNA or the consensus 23S rRNA models [14] used for reference. The number of copies of 23S rRNA genes present in a genome was determined by whole genome BLAST search based on the known 23S rRNA sequence.

### Analysis of intragenomic diversity in 23S rRNA genes

Genomes that contained only a single 23S rRNA gene were not further analyzed. Copies of 23S rRNA genes from each remaining genome were aligned with Clustalw [15]. To calculate diversity, the number of revealed substitutions including point mutations and insertions/deletions (indels) was divided by the total number of positions, including gaps in the alignment.

### Comparison of 2° structures

To compare two related 2° structures, a mismatch was defined as conserved if located in a loop, or located in a stem but causing GC:GU conversions or covariation resulting in no change in base-pairing. In contrast, a non-conserved mismatch altered base-pairing. Substitutions also were classified by the position-specific relative variability rate calculated from the consensus 23S rRNA model based on an alignment of 187 bacterial 23S rRNA genes [14]. Positions were classified as variable or non-variable positions, according to the substitution rate relative to the average substitution rate of all sites [14]. The relative substitution rate for a variable position v>1 indicates a substitution rate higher than that averaged for all sites in the 23S rRNA gene analyzed, while a conserved position had a relative substitution rate v<1; uncommon sites are positions occupied in <25% of organisms due to insertions. The expected variability for certain classes of positions was calculated from the consensus models. Differences between expected and observed variability was analyzed by Chi-Square analysis, considered significant if p<0.05.

### Experimental verification of 23S rRNA gene sequences

Representative 23S rRNA gene sequences containing intervening sequences were experimentally verified by sequence-based PCR using allele-specific primers (Table S1). Bacterial genomic DNA was prepared from strains used in the specific bacterial genome projects. The borders of the 23S rRNA gene sequence and inserts were amplified and sequenced. The sequences obtained were compared with sequences of the corresponding 23S rRNA genes from the edited whole genomes to identify discrepancies.

## Results

### rRNA gene database

In total, 342 complete prokaryotic genomes were available for analysis, 27 from Archaea and 316 from Bacteria. Of the 342 genomes, 73 redundant genomes were removed from the database, as were 85 genomes that contained only a single *rrn* operon. Remaining were 184 unique (10 Achaea, 174 Bacteria) species whose genomes contained multiple 23S rRNA genes (Table S2). The 184 species represented 11 phyla, of which Proteobacteria was the most abundant phylum (98 species) followed by Firmicutes (43 species), Euryarchaeota (10 species), Actinobacteria (9 species) (Table S2). The remaining 7 phyla were represented by only 24 species.

### Diversity of 23S rRNA genes in the primary structure

**Figure 1:**
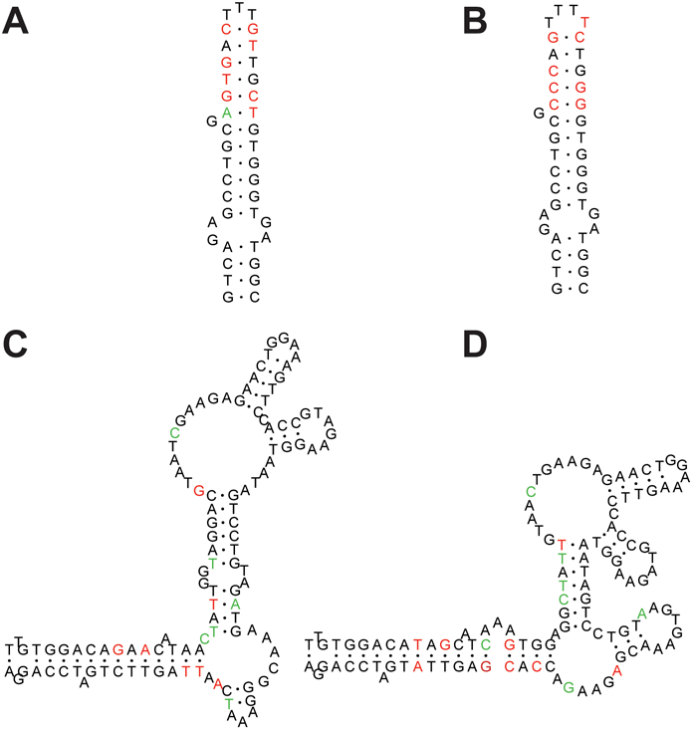
Conservation of 2° structure by complex rearrangement of base pairing and substitutions in *rrn23S* of *Nocardia farcinica* (A and B) and *Clostridium perfringens* (C and D). Nucleotides related to substitutions are highlighted in red and indels in green. Segments of *rrn23S* of *Nocardia farcinica* shown correspond to positions 620 through 659 of *rrnC23S*. The segments of *rrnC23S* (A) and *rrnB23S* (B) differ by 9 positions, including one indel and eight substitutions. Segments of *rrn23S* of *Clostridium perfringens* shown correspond to positions 292 through 406 of *rrnH23S*. The segments of *rrnH23S* (C) and *rrnD23S* (D) differ by 22 positions, including 13 indels and 8 substitutions.

**Figure 2:**
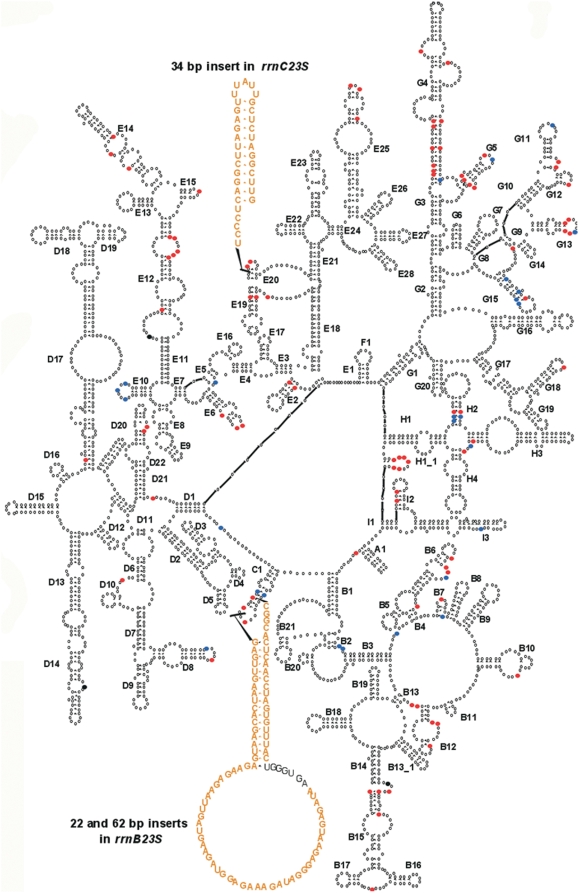
Distribution of substituted positions and IVS in 23S rRNA genes of *T. tengcongensis*. Secondary structure of *rrnB16S* and *rrnB23S* was based on the 2° structure models for *Thermus thermophilus* [14]. Substituted positions between *rrnB23S* and *rrnC23S* are highlighted in colors according to the position-specific relative variability rate calculated from the consensus *rrn23S* model based on an alignment of 184 bacterial 23S rRNA genes [14]. A position with a relative substitution rate v>1 (red) implies that it has a substitution rate higher than the average substitution rate of all its sites in the rRNA gene analyzed, while v<1 (blue) indicates that the rate is lower than the average rate. Uncommon sites are positions that are occupied in <25% of organisms because of insertions, which are shown by black dots.

The 184 genomes had a median of 4.57 23S rRNA genes/genome (range 1 to 15). Diversity was found in 113 genomes, with mean 0.40% and range from 0.01% to 4.04% (median 0.24%, interquartile range (IQR) 0.10%–0.52% sequence variation). The threshold for identification of outliers, calculated as IQR±1.5 IQR, was 0–1.15%. Using this threshold, 8 genomes were identified as having high intragenomic variation amongst their paralogous 23S rRNA genes (Table 1). The eight outliers were concentrated in the four most abundant phyla, four in Proteobateria, two in Firmicutes, one in Actinobacteria, and one in Euryarchaeota. The absence of outliers in the remaining seven phyla is likely because they were least represented in the dataset. In 4 genomes, *Carboxydothermus hydrogenoformans*, *Haloarcula marismortui*, *Shewanella oneidensis*, and *Streptococcus pyogenes*, there was on average 1.52% diversity due to 44.6 substitutions including 8.8 indels (Table 1). In 3 genomes, *Nocardia farcinica* (1.18% diversity, 37 point mutations, 1 indel), *Clostridium perfringens* (1.96% diversity, 16 point mutations, 41 indels), and *Salmonella typhimurium* (1.27% diversity, 16 point mutations, 41 indels), substitutions tended to concentrate within short segments of 23S rRNA genes causing complex rearrangement of the secondary structure (Figure 1), which will be described in detail separately. Highest diversity was observed in *Thermoanaerobacter tengcongensis* (4.04% diversity, 107 point mutations, 8 indels) that also contained intervening sequences (IVS) (Figure 2), which will be described below.

**Table 1:** Genomes with significant intragenomic diversity among paralogous 23S rRNA genes.

Organism	Accession number	Copy	Alignment (nt)	Diversity (%)	Substitution*	Covariation	G:U = G:C	In-loop	Stem-loop transition	Indel
*Carboxydothermus hydrogenoformans*	CP000141	4	2921	1.30	38	4	2	25	7	2
*Haloarcula marismortui*	AY596297	3	2922	1.54	45	24	2	11	8	0
*Shewanella oneidensis*	AE014299	9	2894	1.17	34	22	4	5	3	1
*Streptococcus pyogenes*	AE004092	6	2912	1.65	48	22	8	11	7	8
Average		6.4	2912	1.52	44.6	14.4	4	14.4	8.2	8.8
*Nocardia farcinica*	AP006618	3	3132	1.18	37	Complex rearrangement**				1
*Clostridium perfringens*	BA000016	10	2915	1.96	57					41
*Salmonella typhimurium*	AE006468	7	3093	1.27	39					3
*Thermoanaerobacter tengcongensis*	AE008691	4	2965	4.04	115	24	19	62	10	8

* Substitution includes both point mutations and indels. **See Figure 1.

### Effect on secondary structure

In *T. tengcongensis*, compared with the consensus 2° structure model of 23S rRNA [14] (Figure 2), 86 (74.8%) of the 115 mismatch positions occurred at highly variable positions - significantly higher than expected (40.4%, p<0.0001) (Table 2). Comparing the 2° structures of *rrnB23S* and *rrnC23S*, 105 of the 115 substitutions were conservative (62 in loops [including 4 insertions], 24 covariations, and 19 GU:GC conversions). Only 10 (9 point mutations and 1 single nucleotide insertion) of the 115 substitutions altered the 2° structure by causing stem-loop transition. These features indicate strong structural constraints, suggesting that *rrnC23S* may be functional.

**Table 2:** Observed and expected variability in 23S rRNA genes in *T. tengcongensis.*

Relative variability(i)	Observed substitution rate (%)	Expected substitution rate(%)	P value
i>1	78.4 (86/115)	40.4 (1175/2912)	<0.001
I<1	22.6 (26/115)	58.6 (1707/2912)	<0.001
Uncommon sites	2.6 (3/115)	1.0 (30/2912)	0.110

Besides *T tengcongensis*, conservation of the 2° structure also is apparent despite changes in the primary structure (Table 1) in other species. In *C. hydrogenoformans*, *H. marismortui*, *S. oneidensis*, and *S. pyogenes*, there are on average 44.6 substitutions per genome, of which only ∼20% (8.2 substitutions) may potentially cause stem-loop transition. The effect of other substitutions are minimized by either covariations (14.4 substitutions), G:U to G:C transitions (4 substitutions) or in-loop substitutions (14.4). Complex rearrangements of the 2° structure likely occurred in *N. farcinica*, *C. perfringens*, and *S. typhimurium* as a result of changes in the primary structure (Figure 1). The complex rearrangement appears to be associated with indels. One example is between *rrnC23S* and *rrnB23S* of *Nocardia farcinica* in which a single deletion accompanied 8 point mutations in a segment of 40 nt. The topographic structure can be conserved by repositioning the bases *rrnB23S* although the resultant stem is one base pair shorter and the loop is one base larger (Figure 1A and 1B). Another example is the *rrnH23S* and *rrnD23S* of *C. perfringens* in which there are 13 indels and 8 point mutations in a segment of 115 nt. Although there was an apparent change in the 2° structure, rearrangement of the positions of the loops and the length of the stems appear to have minimized the effect on the topographic structure (Figure 1C and 1D). Variation of *rrn23S* in *S. typhimurium* has been previously reported [16].

### Intervening sequences

Another type of alteration of 23S rRNA genes is insertion of an intervening sequence (Table 3). IVS was found in 7 genomes (Table 3). If the IVS are not included in calculations, the remaining sequences of paralogous 23S rRNA genes have <1% diversity in 6 of the 7 genomes except for *T. tengcongensis* whose diversity is due to both IVS-like insertions and a high level of substitutions.

**Table 3:** Genomes with intervening sequences in 23S rRNA genes.

Species	Accession #	No. of copies	Length Range (nt)	IVS (nt)	Diversity %	Type of IVS
*Agrobacterium tumefaciens*	AE007869.2	4	2233–2252	9,13	0.67	Short IVS
*Deinococcus radiodurans*	AE000513.1	3	2877–3944	1067	0.07	IVS encoding transposon
*Nostoc sp.*	BA000019.2	4	2828–4299	1471	0	IVS encoding transposon
*Photobacterium profundum*	CR354531.1	15	2894–2926	14,17	0.83	Short IVS
*Photorhabdus luminescens*	BX470251.1	7	2909–3021	112	0.96	IVS
*Salmonella typhimurium*	AE006468.1	7	2994–3093	83, 99	1.27	IVS
*Thermoanaerobacter tengcongensis*	AE008691.1	4	2881–2965	22, 62, 34	4.04	IVS and mutations

#### Transposon-mediated insertion of IVS

**Figure 3:**
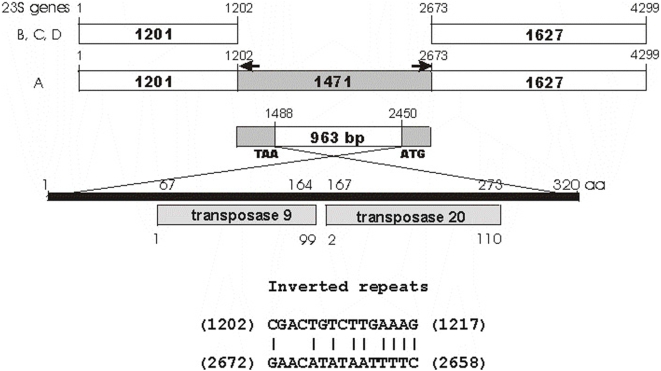
IVS in *Nostoc* sp (strain PCC 7120) 23S rRNA genes. A 1471-bp IVS flanked by partial 16-bp inverted repeats (arrows) was found in *rrnA23S*, which contains a 963-bp open reading frame that encodes proteins with strong homology (Expected value = 7e^−27^) to transposases 9 and 20 in COG3547.

*Nostoc* sp PCC7120 contained four 23S rRNA genes*; rrnA23S* is 4299-nt, while the other three each are 2828-nt [17]. The variation was due to the presence of a 1471-nt insert in *rrnA23S* (Figure 3), including 15-nt imperfect inverted repeats at both ends of the insertion and a 963-nt ORF. The deduced protein is predicted to be a transposase in Clusters of Orthologous Group (COG) COG3547 (Expected value = 7e^−27^). The 303-amino acid of COG3547 consensus consists of both transposases 9 and 20. Blast of the 1471-nt insert against the genome of *Nostoc* sp PCC7122 revealed 11 other homologs, each with the terminal inverted repeats. Without the insertion, all four *rrn*23S copies were identical.

**Figure 4:**
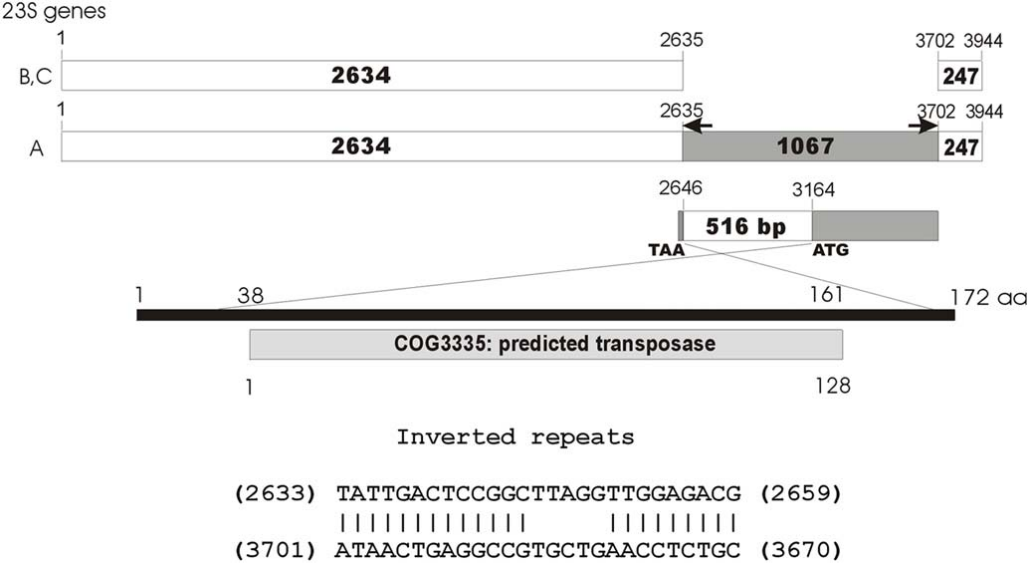
IVS in *Deinococcus radiodurans* 23S rRNA genes. *rrnA23S* contains a 1067-bp IVS flanked by 27-bp inverted repeats. The arrows indicate the location of the inverted repeats. The 1067-bp insert contains a 516-bp open reading frame encoding a 172-amino acid protein with strong homology (Expected value = 8e^−14^) to predicted transposase COG3335.

The 23S rRNA genes of *Deinococcus radiodurans* were only partially annotated (GenBank accession: AE000513) [18]. The full-length 23S rRNA genes were identified by search of the *D. radiodurans* genome using the unassigned RNA (GenBank accession:30749903) associated with the 50S ribosomal subunit [19]. Three putative 23S rRNA genes were identified. The 2° structures of the putative 23S rRNA genes were compared with the consensus prokaryotic 23S rRNA model to confirm identity and to define length [14]. *rrnC23S* was 3944-nt while *rrnA23S* and *rrnB23S* were only 2877-nt (Figure 4). *rrnC23S* contains a 1067-nt insertion including a 519-nt ORF encoding a putative172-amino acid protein. The protein was predicted to be a transposase in COG3335 (Expected value = 8e^−14^) and 27-nt imperfect inverted repeats were present at both ends of the insert. BLAST of the 1067-nt insert *against the D. radiodurans* genome revealed two other nearly identical (>98% homology) copies, including the inserted repeats, located on plasmids MP1 and CP1. *rrnA23S* and *rrnB23S* were nearly identical. Excluding the insertion, only six single-nucleotide substitutions were observed between the three 23S rRNA genes.

#### Small IVS

*Salmonella typhimurium* has seven 23S rRNA genes with sizes ranging from 2994 to 3093 bp [20]. Two insertions were found, 83-nt and 99-nt, consistent with previously described IVS in *S. typhimurium* [16] (Table 3). The largest 23S rRNA gene contains both inserts. The second largest 23S rRNA gene contains the 98-nt insert. The remaining five 23S rRNA genes only contain the 83-nt insert. The largest difference (1.27%, 37/2903) was found between *rrnB23S* and *rrnF23S* with 37 substitutions (Table 1).

*Photorhabdus luminescens* contains seven 23S rRNA genes [21]. *rrnC23S* and *rrnE23S* contain a shared 112-nt insert (Table 3). The other five 23S rRNA genes are 2909 nt in length. The insert is similar to those from *S. typhimurium* in size and in that it does not contain an ORF. No repetitive DNA was present in or near the insertion. Excluding the insert, the largest difference was observed between *rrnD23S* and *rrnE23S*, differing by 28 nt (0.96%). No sequences homologous to the insert were found in *P. luminescens* or GenBank.

*Photobacterium profundum* contains 15 paralogous 23S rRNA genes ranging from 2893–2926 nt in size (GeneBank accession: CR354531.1, unpublished). The size variation was caused by two IVS of 14 and 17 nt, respectively. Maximal diversity was 0.83% (24/2893) between *rrnC23S* and *rrnJ23S* (Table 3). No additional copy of the IVS was found outside of the 23S rRNA genes within the *P. profundum* genome.

*Agrobacterium tumefaciens* contains four copies of 23S rRNA genes, two on each of its linear and circular chromosomes, respectively [22]. The sizes of the 23S rRNA genes vary between 2233 and 2252 nt because the second 23S rRNA gene is shorter, caused by two small IVS of 9 and 13 nt, respectively (Table 3). Besides the IVS, 15 mutations were observed (0.67% diversity).

#### IVS and point mutations

*T. tengcongensis* had four 23S rRNA genes with sizes ranging from 2881 to 2965-nt, reflecting 3 major alleles [23]. The largest difference was between *rrnB23S* and *rrnC23S*, due to inserts of 22, 62, and 34-nt (Figure 2, Table 1) and 115 substitutions. The 22 and 62-nt inserts were present in *rrnA23S* and *rrnB23S*, respectively, the 34-nt insert in *rrnC23S*, and none in *rrnD23S*. No sequences homologous to the three inserts were found in GenBank. Excluding the 3 inserts, there remained differences of 4.0% (115/2884) between *rrnB23S* and *rrnC23S*.

### Verification of 23S rRNA gene sequences by PCR-based sequencing

To examine the reliability of the whole genome sequences, we experimentally verified the IVS of *T. tengcongensis*, *D. radiodurans*, and *Nostoc sp*. The predicted junctions between the rRNA sequences and the IVS were present in all three strains tested. The sequences of cloned PCR products are identical to those published in the genome studies, providing validation of the presence of the diversity observed in the whole genome sequences. Because we did not experimentally verify the IVS of all species, our results are limited, although in total, the data suggest that the whole genome sequences are highly reliable.

## Discussion

This is the first large scale survey of intragenomic variation of paralogous 23S rRNA genes. We analyzed 23S rRNA genes from genomes representing 184 prokaryotic species to examine for evidence of ribosomal constraint of rRNA structures at the intragenomic level. Our findings support the hypothesis that individual 23S rRNA genes within a genome are conserved due to such structural constraints.

One level of ribosomal constraint on 23S rRNA genes was observed at the level of primary structure. In the large majority of the genomes (183/184) analyzed, variation of primary structure among paralogous 23S rRNA genes was small (<3%, which is the 16S *rrn*-based species boundary) [24]. In our dataset, 38% of the 184 genomes containing multiple paralogous 23S rRNA genes were invariable, similar to the finding in 16S rRNA genes [25]. Our findings of 0.4% average diversity for intragenomic variation of rRNA sequences among the 113 diversified genomes is higher than the reported 0.17% diversity among 16S rRNA genes [26], but will be more similar to the 16S rRNA genes diversity (0.25%) if all 184 genomes are included in the calculation (Table S2). Excluding *T. tengcongensis*, which has unusually high diversity among 23S rRNA genes, the maximal diversity among paralogous 23S rRNA genes is 1.96%, higher than the maximal variation of 1.26% among 16S rRNA genes [27]. In organisms containing multiple rRNA genes, the homogeneity of primary sequences is believed to be maintained through gene conversion by homologous recombination [2], [28]. The evolution of these paralogous rRNA genes is concerted in such a fashion that within a species the rRNA genes are nearly identical, whereas orthologous rRNA genes between species can vary considerably [2], [28]. However, our study suggests that homologous recombination is not always efficient in keeping the primary structure in check. In *T. tengcongensis*, the 4% difference (without considering the IVS) in primary structure between *rrnB23S* and *rrnC23S* is in agreement with the high % divergence reported between 16S rRNA paralogues [25]. It is tempting to attribute the unusual variation to the presence of IVS in the *T. tengcongensis* 23S rRNA genes, but variation amongst 23S rRNA genes in six other genomes containing IVS is much lower (Table 3). It appears that gene conversion or concerted evolution is one, but not the only, way to meet the requirement of ribosomal constraint.

Another level of ribosomal constraint was observed at the level of 2° structures. For example, the detailed analysis of the 23S rRNA genes of *T. tengcongensis* showed only in uncommon instance (10 of 115 point substitutions) a point substitution altered the 2° structure. This observation is in line with the current concept derived from comparing consensus rRNA sequences from closely related species [29], [30], [31], [32], [33] in that selection pressure from ribosomal constraints is to preserve 2° structures that are essential for the assembly of functional ribosomes [34]. In organisms with a single rRNA operon, ribosomal constraints could follow a simple birth-and-death model by linking the fate of a mutation to the viability of the organisms. However, in an organism with multiple rRNA operons, the nature of the selection pressure is unclear. In *Escherichia coli*, which has seven rRNA operons, after deletion of the other 6 operons only a single operon is sufficient to produce >50% of wild-type rRNA levels and support >50% of the wild-type growth rate [35]. This experiment suggests that in an organism with multiple rRNA operons, it may not be necessary for survival to keep all rRNA genes under ribosomal constraint. The observed tight constraints on individual 23S rRNA in *T. tengcongensis* needs a new explanation. *T. tengcongensis*, isolated from a fresh water hot spring in Tengchong, China, is a rod-shaped, gram-negative bacterium that grows anaerobically in extreme environments. It propagates at wide ranges of temperatures from 50° to 80°C and pH values between 5.5 and 9 [23]. It is possible that all four 23S rRNA genes are essential for survival of *T. tengcongensis* in its natural inhabit, though one might be sufficient under laboratory conditions. It also is possible that the divergence of 23S rRNA genes offers competitive advantage in a changing environment in nature and thus are selectively maintained. Ribosomal constraints could be met by maintaining conserved 2° structures through conserved point mutations under sufficient selective pressure to resist the tide of homologous recombination.

Our finding is consistent with the finding by Acinas *et al.* in *rrnC16S* of *T. tengcongensis* [25]. Both studies indicate that the 2° structures are conserved despite a high level of change in the primary structures and suggest that the *rrnC* operon is functional. Although Acinas *et al*. suggested that the rrnC operon could be brought into *T. tengcongensis* by horizontal gene transfer, the hypothetic donor has yet to be identified. The ribosomal constraint observed at the level of 2° structures is also evident in 23S rRNA genes in *C. hydrogenoformans*, *H. marismortui*, *S. oneidensis*, and *S. pyogenes*.

Complex rearrangement of the position of nucleotides, the position and size of loops, and the length of stems might be another mechanism to conserve the functional structure of an rRNA gene. The examples shown in *N. farcinica* and *C. perfringens* are beyond simple alteration or conservation of the 2° structures since there is no straightforward way to quantify the changes and their effects. Rather, this type of rearrangement seems to aim at conserving the topographic structure in the presence of drastic changes in the primary and secondary structures. However, an experimental approach will be required to address the relationship between the conservation of the topographic structure and function of an rRNA gene.

The concept of rRNA conservation has revolutionized exploration of microbial ecosystems and identifying the causes of diseases of unknown etiologies [36], [37], [38], [39]. The most common molecular sequences used for phylogenetic analysis are ribosomal RNAs. The 16S rRNA genes have become the most widely used sequences for phylogenetic analysis as seen by the vast and growing database of 16S rRNA genes [40], [41]. The choice of 16S rRNA as the most widely used sequences for phylogenetic analysis has been based on the simple assumption that 16S rRNA is optimal because 5S rRNA is too short while 23S rRNA sequencing requires extra effort without a commensurate increase in phylogenetic signal. However, misidentification has been reported. Strains from different species may have identical 16S rRNA gene sequences, and strains of one species may have 16S rRNA that differ by >4% while 3% is considered as the threshold for within-species dissimilarity [42], [43], [44]. On the other hand, the main obstacle limiting 23S rRNA from being used in classification of microorganisms has significantly diminished, as the cost for DNA sequencing is becoming less expensive. Evaluation of intragenomic variation of the rRNA genes has become possible since more and more sequences of whole microbial genomes have recently become available. It would be a logical expectation that the intragenomic diversity of a marker for taxonomic classification of microorganisms is below the level of interspecies diversity. We evaluated the suitability of 23S rRNA genes in this regard. Our study indicates that high-level variation among paralogous 23S rRNA genes might exceed the level of variation among 23S rRNA genes from different strains within a species but will not have a significant effect on the integrity of a 23S rRNA-based phylogenetic system, because its occurrence was rare. The insertions encountered when using 23S rRNA genes in phylogenetic analysis can be easily recognized and eliminated in comparisons of homologous sequences.

Intervening sequences (IVS) were frequently observed in 23S rRNA genes. In the seven species in which IVS were identified, five species contain small IVS that did not contain any ORFs but in *D. radiodurans* and *Nostoc* sp, IVS were large and contained ORFs encoding putative transposases. Experimental studies have demonstrated that IVS in 23S rRNA does not interfere with its functions[45]. The 23S rRNA of *Salmonella typhimurium* LT2 are known to carry IVS at two sites, helix-25 and helix-45, which are excised by RNase III during rRNA maturation, resulting in rRNA which is fragmented, but nevertheless functional [45]. Fragmentation of the 23S rRNA also has been observed in diverse Eubacteria [46]. 23S rRNAs with insertions have been shown to rescue an *E. coli* strain in which all chromosomal rRNA operons were deleted [47].

## Supporting Information

Table S1 Bacterial strains and primers used in verification of IVS in 23S rRNA genes.
(0.04 MB DOC)

Table S2 Intragenomic diversity of 23S rRNA genes in Bacteria and Archaea.
(0.26 MB DOC)
