# Supplementary material for: Diversity of 23S rRNA Genes within Individual Prokaryotic Genomes
Source: PLoS One. 2009 May 5;4(5):e5437. doi: 10.1371/journal.pone.0005437 (PMC2672173; doi:10.1371/journal.pone.0005437)
Supplement: Table S1 — Bacterial strains and primers used in verification of IVS in 23S rRNA genes. (0.04 MB DOC) [file pone.0005437.s001.doc]

| Table S1. Bacterial strains and primers used in verification of IVS in 23S rRNA genes | | | |
| --- | --- | --- | --- |
| Species (Strain) | GenBank accession | Primers (5’ to 3’) | Target |
| *D.radiodurans* (R1) | AE000513.1 | 2F attaaagcggcacgcgagctg | Wild type 23S rRNA gene |
|  |  | 2R gttccggacatagctaccctg |  |
|  |  | 1F tttggcacctcgatgtcggct | 5’ border of IVS |
|  |  | 1R cacgtccaaggcaagctcgt |  |
| *Nostoc sp.* (PCC 7120) | AE000513.1 | 1F gaggtttgcctagaagcagcc | 5’ border of IVS |
|  |  | 1R gtctttttgcccgtaccagcc |  |
|  |  | 1F tgcctacccacacagagatgtt | 3’ border of IVS |
|  |  | 1R gtgtccatcgactacgccttt |  |
| *T.tengcongensis* (MB1) | AE008691.1 | 3F cgccctcttcggactcgct | IVS |
|  |  | 3R gtaccacgggataggcgacc |  |
