# Supplementary material for: Diversity of 23S rRNA Genes within Individual Prokaryotic Genomes
Source: PLoS One. 2009 May 5;4(5):e5437. doi: 10.1371/journal.pone.0005437 (PMC2672173; doi:10.1371/journal.pone.0005437)
Supplement: Table S2 — Intragenomic diversity of 23S rRNA genes in Bacteria and Archaea. (0.26 MB DOC) [file pone.0005437.s002.doc]

| Table S2. Intragenomic diversity of 23S rRNA genes in Bacteria and Archaea | | | |
| --- | --- | --- | --- |
| Organism | | Number of copies | % Diversity |
| **Bacteria: Actinobacteria (n = 9)** | | | |
| *Bifidobacterium longum* | | 4 | 0.20 |
| *Corynebacterium diphtheriae* | | 5 | 0.10 |
| *Corynebacterium efficiens* | | 5 | 0.01 |
| *Corynebacterium glutamicum* | | 6 | 0.16 |
| *Corynebacterium jeikeium* | | 3 | 0.33 |
| *Frankia sp.* | | 2 | 0 |
| *Nocardia farcinica* | | 3 | 1.18 |
| *Propionibacterium acnes* | | 3 | 0.01 |
| *Thermobifida fusca* | | 4 | 0.03 |
| Average | | 3.9 | 0.22 |
|  | |  |  |
| **Bacteria: Aquificae (n = 1)** | | | |
| *Aquifex aeolicus* | | 2 | 0 |
| Average | | 2 | 0 |
|  | |  |  |
| **Bacteria: Bacteroidetes (n = 5)** | | | |
| *Bacteroides fragilis* | | 6 | 0.35 |
| *Bacteroides thetaiotaomicron* | | 5 | 0.98 |
| *Chlorobium tepidum* | | 2 | 0 |
| *Pelodictyon luteolum* | | 2 | 0.07 |
| *Porphyromonas gingivalis* | | 4 | 0 |
| Average | | 3.8 | 0.28 |
|  | |  |  |
| **Bacteria: Chlamydiae (n = 3)** | | | |
| *Chlamydia muridarum* | | 2 | 0.03 |
| *Chlamydia trachomatis* | | 2 | 0.10 |
| *Parachlamydia sp.* | | 2 | 0.14 |
| Average | | 2.0 | 0.09 |
|  | |  |  |
| **Bacteria: Cyanobacteria (n = 6)** | | | |
| *Anabaena variabilis* | | 4 | 0 |
| *Nostoc sp.* | | 4 | 0 |
| *Prochlorococcus marinus* | | 2 | 0 |
| *Synechococcus elongatus* | | 2 | 0 |
| *Synechococcus sp.* | | 2 | 0.10 |
| *Synechocystis sp.* | | 2 | 0 |
| Average | | 2.7 | 0.02 |
|  | |  |  |
| **Bacteria: Deinococcus-Thermus (n = 3)** | | | |
| *Deinococcus geothermalis* | | 3 | 0.21 |
| *Deinococcus radiodurans* | | 3 | 0.07 |
| *Thermus thermophilus* | | 2 | 0 |
| Average | | 2.7 | 0.09 |
|  | |  |  |
| **Archaea: Euryarchaeota (n = 10)** | | | |
| *Haloarcula marismortui*^a^ | | 3 | 1.54 |
| *Methanocaldococcus jannaschii*^a^ | | 2 | 0.24 |
| *Methanococcoides burtonii*^a^ | | 3 | 0.10 |
| *Methanococcus maripaludis*^a^ | | 3 | 0 |
| *Methanosarcina acetivorans*^a^ | | 3 | 0.14 |
| *Methanosarcina barkeri*^a^ | | 3 | 0.24 |
| *Methanosarcina mazei*^a^ | | 3 | 0.24 |
| *Methanosphaera stadtmanae*^a^ | | 4 | 0.24 |
| *Methanospirillum hungate*^a^*i* | | 4 | 0.24 |
| *Methanothermobacter thermautotrophicus*^a^ | | 2 | 0.53 |
| Average | | 3.0 | 0.35 |
|  | |  |  |
| **Bacteria: Firmicutes (n = 43)** | | | |
| *Aster yellows witches'-broom phytoplasma* | | 2 | 0.14 |
| *Bacillus anthracis* | | 11 | 0.41 |
| *Bacillus cereus* | | 13 | 0.43 |
| *Bacillus clausii* | | 7 | 0.51 |
| *Bacillus halodurans* | | 8 | 0.10 |
| *Bacillus licheniformis* | | 7 | 0.58 |
| *Bacillus subtilis* | | 10 | 0.92 |
| *Bacillus thuringiensis* | | 14 | 0.17 |
| *Carboxydothermus hydrogenoformans* | | 4 | 1.30 |
| *Clostridium acetobutylicum* | | 11 | 0.28 |
| *Clostridium perfringens* | | 10 | 1.96 |
| *Clostridium tetani* | | 6 | 0.21 |
| *Desulfitobacterium hafniense* | | 6 | 0.68 |
| *Enterococcus faecalis* | | 4 | 0.10 |
| *Geobacillus kaustophilus* | | 9 | 0.41 |
| *Lactobacillus johnsonii* | | 6 | 0 |
| *Lactobacillus plantarum* | | 5 | 0.21 |
| *Lactobacillus sakei* | | 7 | 0.10 |
| *Lactobacillus salivarius* | | 7 | 0.39 |
| *Lactococcus lactis* | | 6 | 0.01 |
| *Listeria innocua* | | 6 | 0.71 |
| *Listeria monocytogenes* | | 6 | 0.68 |
| *Mesoplasma florum* | | 2 | 0 |
| *Mycoplasma capricolum* | | 2 | 0.14 |
| *Mycoplasma gallisepticum* | | 2 | 0.10 |
| *Mycoplasma mycoides* | | 2 | 0.48 |
| *Mycoplasma synoviae* | | 2 | 0.35 |
| *Oceanobacillus iheyensis* | | 7 | 0.17 |
| *Onion yellows phytoplasma* | | 2 | 0 |
| *Staphylococcus aureus* | | 5 | 0.11 |
| *Staphylococcus epidermidis* | | 5 | 0.32 |
| *Staphylococcus haemolyticus* | | 5 | 0.28 |
| *Staphylococcus saprophyticus* | | 6 | 0.14 |
| *Streptococcus agalactiae* | | 7 | 0 |
| *Streptococcus mutans* | | 5 | 0 |
| *Streptococcus pneumoniae* | | 4 | 0 |
| *Streptococcus pyogenes* | | 6 | 1.65 |
| *Streptococcus thermophilus* | | 6 | 0.20 |
| *Streptomyces avermitilis* | | 6 | 0.80 |
| *Streptomyces coelicolor* | | 6 | 0.74 |
| *Symbiobacterium thermophilum* | | 6 | 0.41 |
| *Thermoanaerobacter tengcongensis* | | 4 | 4.04 |
| *Ureaplasma parvum* | | 2 | 0 |
| Average | | 6.0 | 0.47 |
|  | |  |  |
| **Bacteria: Fusobacteria (n = 1)** | | | |
| *Fusobacterium nucleatum* | | 5 | 0.19 |
| Average | | 5.0 | 0.19 |
|  | |  |  |
| **Bacteria: Proteobacteria (n = 98)** | | | |
| *Acinetobacter sp.* | | 7 | 0 |
| *Agrobacterium tumefaciens* | | 4 | 0.67 |
| *Anaeromyxobacter dehalogenans* | | 2 | 0.03 |
| *Azoarcus sp.* | | 4 | 0.03 |
| *Bartonella henselae* | | 2 | 0 |
| *Bartonella quintana* | | 2 | 0 |
| *Baumannia cicadellinicola* | | 2 | 0 |
| *Bdellovibrio bacteriovorus* | | 2 | 0 |
| *Bordetella bronchiseptica* | | 3 | 0 |
| *Bordetella parapertussis* | | 3 | 0 |
| *Bordetella pertussis* | | 3 | 0 |
| *Brucella abortus biovar 1* | | 3 | 0 |
| *Brucella melitensis* | | 6 | 0.39 |
| *Brucella suis* | | 3 | 0.99 |
| *Burkholderia mallei* | | 3 | 0 |
| *Burkholderia pseudomallei* | | 4 | 0.07 |
| *Burkholderia sp.* | | 6 | 0.28 |
| *Burkholderia thailandensis* | | 4 | 0.03 |
| *Burkholderia xenovorans* | | 6 | 0 |
| *Campylobacter jejuni* | | 3 | 0 |
| *Caulobacter crescentus* | | 2 | 0 |
| *Chromobacterium violaceum* | | 8 | 0 |
| *Chromohalobacter salexigens* | | 5 | 0.24 |
| *Colwellia psychrerythraea* | | 9 | 0.24 |
| *Dechloromonas aromatica* | | 4 | 0 |
| *Desulfotalea psychrophila* | | 7 | 0.65 |
| *Desulfovibrio desulfuricans* | | 4 | 0.07 |
| *Desulfovibrio vulgaris* | | 5 | 0.01 |
| *Erwinia carotovora* | | 7 | 0.14 |
| *Escherichia coli* | | 7 | 0.72 |
| *Francisella tularensis* | | 3 | 0 |
| *Geobacter metallireducens* | | 2 | 0.07 |
| *Geobacter sulfurreducens* | | 2 | 0 |
| *Gluconobacter oxydans* | | 4 | 0.15 |
| *Haemophilus ducreyi* | | 6 | 0 |
| *Haemophilus influenzae* | | 6 | 0 |
| *Hahella chejuensis* | | 5 | 0 |
| *Helicobacter pylori* | | 2 | 0.10 |
| *Idiomarina loihiensis* | | 4 | 0.47 |
| *Lawsonia intracellularis* | | 2 | 0 |
| *Legionella pneumophila* | | 3 | 0.07 |
| *Magnetospirillum magneticum* | | 2 | 0 |
| *Mannheimia succiniciproducens* | | 6 | 0.17 |
| *Mesorhizobium loti* | | 2 | 0 |
| *Methylobacillus flagellatus* | | 2 | 0 |
| *Methylococcus capsulatus* | | 2 | 0 |
| *Neisseria gonorrhoeae* | | 4 | 0.52 |
| *Neisseria meningitidis* | | 4 | 0 |
| *Nitrosococcus oceani* | | 2 | 0 |
| *Novosphingobium aromaticivorans* | | 3 | 0 |
| *Pasteurella multocida* | | 6 | 0.31 |
| *Pelobacter carbinolicus* | | 2 | 0 |
| *Photobacterium profundum* | | 15 | 0.83 |
| *Photorhabdus luminescens subsp. laumondii* | | 7 | 0.96 |
| *Pseudoalteromonas haloplanktis* | | 9 | 0.17 |
| *Pseudomonas aeruginosa* | | 4 | 0.01 |
| *Pseudomonas entomophila* | | 7 | 0.28 |
| *Pseudomonas fluorescens* | | 6 | 0 |
| *Pseudomonas putida* | | 7 | 0.17 |
| *Pseudomonas syringae* | | 5 | 0.01 |
| *Psychrobacter arcticus* | | 4 | 0 |
| *Psychrobacter cryohalolentis* | | 4 | 0.04 |
| *Ralstonia eutropha* | | 6 | 0.39 |
| *Ralstonia metallidurans* | | 4 | 0.04 |
| *Ralstonia solanacearum* | | 3 | 0 |
| *Rhizobium etli* | | 3 | 0 |
| *Rhodobacter sphaeroides* | | 2 | 0 |
| *Rhodoferax ferrireducens* | | 2 | 0 |
| *Rhodopseudomonas palustris* | | 2 | 0 |
| *Rhodospirillum rubrum* | | 4 | 0.11 |
| *Saccharophagus degradans* | | 2 | 0 |
| *Salmonella typhi* | | 7 | 0.27 |
| *Salmonella typhimurium* | | 7 | 1.27 |
| *Shewanella denitrificans* | | 8 | 0.55 |
| *Shewanella oneidensis* | | 9 | 1.17 |
| *Shigella boydii* | | 7 | 0.59 |
| *Shigella dysenteriae* | | 7 | 0.07 |
| *Shigella flexneri* | | 7 | 0 |
| *Shigella sonnei* | | 7 | 0.38 |
| *Silicibacter pomeroyi* | | 3 | 0 |
| *Sinorhizobium meliloti* | | 3 | 0 |
| *Sodalis glossinidius* | | 7 | 0 |
| *Thiobacillus denitrificans* | | 2 | 0 |
| *Thiomicrospira crunogena* | | 3 | 0.03 |
| *Thiomicrospira denitrificans* | | 4 | 0.03 |
| *Vibrio cholerae* | | 8 | 0.80 |
| *Vibrio fischeri* | | 12 | 0.41 |
| *Vibrio parahaemolyticus* | | 11 | 0.86 |
| *Vibrio vulnificus* | | 9 | 0.59 |
| *Wigglesworthia brevipalpis* | | 2 | 0 |
| *Wolinella succinogenes* | | 3 | 0 |
| *Xanthomonas axonopodis* | | 2 | 0 |
| *Xanthomonas campestris* | | 2 | 0 |
| *Xanthomonas oryzae* | | 2 | 0 |
| *Xylella fastidiosa* | | 2 | 0 |
| *Yersinia pestis* | | 7 | 0.38 |
| *Yersinia pseudotuberculosis* | | 9 | 0.34 |
| *Zymomonas mobilis* | | 3 | 0 |
| Average | | 4.6 | 0.17 |
|  | |  |  |
| **Bacteria: Spirochaetes (n = 5)** | | | |
| *Borrelia burgdorferi* | | 2 | 0.10 |
| *Borrelia garinii* | | 2 | 0 |
| *Leptospira interrogans serovar* | | 2 | 0 |
| *Treponema denticola* | | 2 | 0 |
| *Treponema pallidum* | | 2 | 0 |
| Average | | 2.0 | 0.02 |
|  | |  |  |
| Total (n = 184) | Average | 4.57 | 0.25 |
